# Strengthened General Self-Efficacy with Multidisciplinary Vocational Rehabilitation in Women on Long-Term Sick Leave: A Randomised Controlled Trial

**DOI:** 10.1007/s10926-017-9752-8

**Published:** 2018-01-09

**Authors:** Åsa Andersén, Kjerstin Larsson, Per Lytsy, Erik Berglund, Per Kristiansson, Ingrid Anderzén

**Affiliations:** Department of Public Health and Caring Sciences, Uppsala University, Box 564, 751 22 Uppsala, Sweden

**Keywords:** Sick leave, Vocational rehabilitation, Self-efficacy, Women, Multidisciplinary rehabilitation, Chronic pain, Mental illness

## Abstract

*Purpose* To investigate the effects of two vocational rehabilitation interventions on self-efficacy, for women on long-term sick leave ≥ 1 year due to chronic pain and/or mental illness. *Methods* This study uses data from a randomised controlled trial consisting of two phases and comprising 401 women on long-term sick leave. They were allocated to either (1) a multidisciplinary team assessment and multimodal intervention (TEAM), (2) acceptance and commitment therapy (ACT), or (3) control group. Data were collected through repeated measurements from self-reported questionnaires before intervention, 6 and 12 months later and registry data. Data from measurements of general self-efficacy, sociodemographics, anxiety and depression were analysed with linear regression analyses. *Results* During the intervention period, the women in the TEAM group’s self-efficacy mean increased from 2.29 to 2.74. The adjusted linear regression model, which included group allocation, sociodemographics, self-efficacy pre-treatment, anxiety and depression showed increased self-efficacy for those in the TEAM intervention at 12 months (B = 0.25, 95% CI 0.10–0.41). ACT intervention had no effect on self-efficacy at 12 months (B = 0.02, 95% CI − 0.16 to 0.19). The results in the adjusted model also showed that higher self-efficacy at pre-treatment was associated with a higher level of self-efficacy at 12 months (B = 0.68, 95% CI 0.54–0.81). *Conclusion* A multidisciplinary team assessment and multimodal intervention increased self-efficacy in women on sick leave for an extremely long time (mean 7.8 years) who had a low mean level of self-efficacy prior to inclusion. Thus, self-efficacy needs to be addressed in vocational rehabilitation.

## Introduction

Self-efficacy is a component of social cognitive theory, believed to operate through motivation, actions and thoughts. The concept of self-efficacy was initially outlined by Albert Bandura and is described as being central to human behaviour. Self-efficacy can briefly be described as belief in one’s own ability to handle or perform a specific task or activity and is, thus, related to an individual’s expectations of an outcome. Positive expectations can encourage an action while the reverse can conversely act as an obstacle. Self-efficacy varies in level and strength, and is influenced by an individual’s self-evaluation and experiences [1]. Bandura describes four sources of information from which individuals assess their self-efficacy, these are: (1) verbal persuasion (i.e. social persuasion strengthens the individual’s belief in having enough capacity to manage a specific task); (2) physiological and affective states (i.e. judgment of one’s own ability through the experience of the physical- and mental condition); (3) vicarious experiences (i.e. valuation of one’s own capacity and ability in relation to others); and (4) enactive mastery (i.e. valuation based on earlier successes and failures, successes build a strong belief in one’s own ability and reinforce the level of self-efficacy; in contrast, failures will instead weaken the level of self-efficacy) [1]. Self-efficacy affects the goals that individuals set for themselves and the higher self-efficacy an individual experiences, the greater goals they expect their actions to achieve. Individuals with low self-efficacy will conversely not expect their actions to achieve much and will give up faster if difficulties arise, instead of facing them. According to Bandura, individuals with low self-efficacy needs interactive support and guidance to overcome these obstacles [2]. Although an individual’s self-efficacy influences the direction of their lives, there are other factors of influence [1]. One such factor may be sick leave, which has been shown to affect self-efficacy negatively [3, 4] and may prolong sick leave duration [5].

At the end of 2015, 101,000 women and 51,000 men were on long-term sick leave (≥ 60 days) in Sweden, demonstrating high sickness rates and a gender difference in sick leave utilisation [6]. For the past 30 years, women have been on sick leave more often and for longer periods than men in Sweden and other Western European countries [7, 8]. In 2015, mental health problems represented the most common causes for sick leave in both women (45%) and men (34%), followed by musculoskeletal diseases (19 vs. 22%) [9].

Although sick leave is often necessary for recovery and healing, studies shows that long-term sick leave may have negative side effects by causing a deterioration in health status [10, 11] and by reducing the likelihood of return-to-work (RTW) [12]. In a study by Lannerstrom et al. [13], women on long-term sick leave described how the sick leave initially enabled recovery. However, with time, being on sick leave gave rise to feelings of uncertainty and the awaiting of different events, e.g., medical visits or investigations, resulted in stress and worries. With time, the women on sick leave became more passive, unmotivated and lost their daily routines.

This brought a feeling of powerlessness and individuals who were usually driven started to question their own abilities, illustrating a change in their own self-perception [13]. A study by Hansen and Falkendahl [14], including men and women on sick leave due to different kinds of symptoms/diseases (musculoskeletal-, internal medical- or mental), showed similar results. Participants with low self-efficacy (i.e. low belief in their ability to RTW), remained on sick leave as of a follow-up 2 years after the initial study [14], illustrating that both psychological factors and medical condition need to be considered in vocational rehabilitation as well as early rehabilitation.

The importance of taking psychological factors into consideration in vocational rehabilitation was also highlighted in a recently published review [15], targeting individuals on sick leave due to long-term back, neck and shoulder problems. The review also showed that vocational rehabilitation with a multidisciplinary approach had positive effects on RTW. In multidisciplinary rehabilitation, multiple professions work together in a team. The basis of the team’s work is to plan and coordinate actions to achieve jointly agreed upon goals that are decided in cooperation with the patient. This rehabilitation model is based on a bio-psycho-social perspective, containing a combination of psychological, educational and physical/biological rehabilitation methods, where the psychological actions may consist of, e.g., cognitive behavioural therapy (CBT) [16].

The medical condition is not the sole determinant for RTW after long-term sick leave, also psychosocial factors such as self-efficacy, are important and need to be addressed in vocational rehabilitation [17]. A newly published review article [18] on individuals with common psychological injuries (e.g., depression, stress, anxiety) or musculoskeletal injuries showed that higher levels of self-efficacy had positive associations with RTW outcomes after sick leave. Based on these results, the authors suggested that future studies looking to improve RTW outcomes should focus on improving self-efficacy.

To our knowledge, there are no published studies focusing on the possibility of strengthening self-efficacy through vocational rehabilitation interventions, for individuals who have been on sick leave for a long time, i.e. ≥ 1 year.

The aim of the present study was to investigate whether two vocational rehabilitation interventions result in improved self-efficacy in women on long-term sick leave due to mental illness and/or chronic pain, compared to a control group.

## Method

### Study Design

This study used data from a randomised controlled trial, consisting of two phases, in total comprising 401 Swedish women on long-term sick leave. Data were collected through a registry and repeated measurements from self-report questionnaires, pre-treatment (before intervention) and 6 and 12 months later. The study sample has partly been used and reported in previous studies [19–21].

### Study Population and Procedure

The population consisted of Swedish women on long-term sick leave due to pain and/or mental illness who were expected to reach their maximum time of refunded days (i.e. 365 days within a 450-day period) in the social insurance, according to regulation changes [22]. The Swedish Social Insurance Agency (SSIA) identified 1305 potential participants through registry data. A physician and an occupational therapist or psychologist, screened the potential participants’ medical certificates to meet the inclusion criteria: women, on sick leave because of mental disorder and/or pain, aged 20–64 years and not fulfilling the exclusion criteria: bipolar disorder type 1, schizophrenia, at current suicidal risk, ongoing substance or alcohol abuse (according to the sickness certificates), taking part in psychotherapy or vocational rehabilitation.

**Fig. 1:**
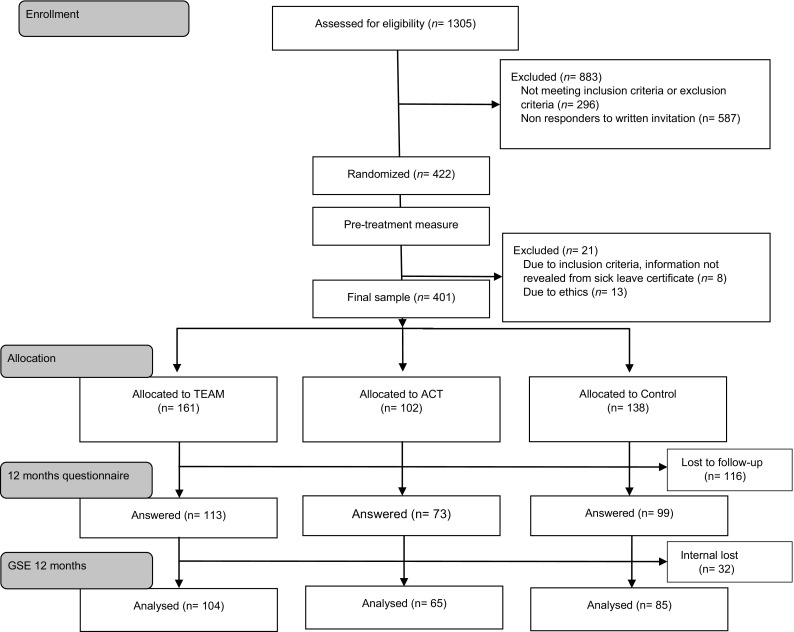
Flow-chart of inclusion- and data collection process

In total, 1009 women fulfilled the inclusion criteria and were invited to take part in the study. In the first phase of the study, 308 women were randomised into one of the following conditions: (a) multidisciplinary team intervention (TEAM; *n* = 102), (b) psychological treatment with acceptance and commitment therapy (ACT; *n* = 102) or (c) control group (*n* = 104). In the second phase of the study, 93 women were randomised into one of the following conditions: (a) TEAM (*n* = 59) or (b) control group (*n* = 34). This change was due to an extension of the TEAM and control group after 1 year. However, in the second phase the ACT intervention was omitted due to reduced inflow of participants in the study and a larger attrition among the participants in the ACT intervention group. The SSIA was responsible for the randomisation. Initially, the participants had an equal chance of being allocated to the following groups: TEAM, ACT or control. After the extension, two-thirds were randomised to the TEAM group and one-third to the control group. After medical assessment, eight women were excluded due to exclusion criteria (information not revealed by sick leave certificate) and 13 due to ethics (excluded from the study because of entering in the intervention prior to ethical approval), leaving 401 women in the final sample allocated to TEAM (161), ACT (102) and control (138), see Fig. 1 for a flow chart of the recruitment- and data collection process.

### The Interventions

There were two intervention groups: (1) multidisciplinary team assessment with a subsequent multimodal intervention (TEAM); and (2) ACT. The interventions were introduced when the participants had 3–4 months remaining before the date when they were expected to reach their maximum time in the social insurance and thereby would be transferred to the Swedish Public Employment Service (SPES). Length of interventions was individualised and could continue over a 12-month period. A detailed description of the interventions is provided elsewhere [21]. The participants in both the TEAM and ACT group received further cooperation between the SSIA, SPES and a designated contact person (i.e. team member or psychologist) participated and took part in the meetings along with the participant.

### Multidisciplinary Team

The multidisciplinary team (TEAM) included a physician, an occupational therapist, a social worker and a psychologist. Each of the TEAM members met the participants separately and performed an assessment of their need for support and rehabilitation based on professional expertise. Thereafter the TEAM members discussed, without the participant, appropriate rehabilitation actions based on the previous assessments, to optimise the individual’s possibility for RTW. The purpose was to develop an individualised rehabilitation plan with suggested interventions. TEAM participants had the possibility to receive ACT if the TEAM proposed it. In total, 88 (55%) of the participants received ACT. Each participant was given a personal contact (one of the TEAM members) who presented the rehabilitation plan to them after the TEAM meeting. The participants could accept either the whole plan or parts of it. The TEAM used weekly meetings to follow up, synchronise actions, and evaluate the rehabilitation. The participants’ mean number of meetings with the various TEAM members was: psychologist (i.e. ACT) 5.0 (SD = 6.6), physiotherapist/occupational therapist 2.0 (SD = 4.1), physician 1.0 (SD = 1.2) and social worker 1.0 (SD = 3.5). After the extension of the study, a physiotherapist was added to the TEAM and the assessments were limited to delivery by a physician and a physiotherapist instead of all TEAM members. No differences were observed in self-efficacy at pre-treatment between the two TEAM groups, i.e. before and after the extension.

The TEAM developed a protocol to assure the quality and procedure for assessment, work procedures and practice. They also got an introduction in ACT, team training and education in motivational interviewing (MI), a communication technique aiming to strengthen an individual’s motivation and commitment to change [23].

### ACT Intervention

The other vocational rehabilitation intervention was a unimodal rehabilitation which included psychologists who used ACT. ACT is a type of CBT, where the intention is to change the individuals’ attitudes to their problems/difficulties based on three main principles: mindfulness, acceptance and fundamental values. Thereby barriers that create limitations in the individual’s life may be removed [24]. The participants in the ACT group only received treatment with ACT. The participant’s mean numbers of ACT sessions with a psychologist was 8.0 (SD = 6.0).

The psychologist in the ACT group and the TEAM group received training in ACT together during the project from a supervisor specialised in ACT. None of the psychologists in the ACT group were working with participants in the TEAM and vice versa.

### Controls

The control group did not receive any collaboration meeting support and went through the usual procedures when transferred from the SSIA to the SPES. Each control participant was followed-up with the same questionnaires as the intervention groups.

## Measurements

Self-reported questionnaire data were collected in all three groups before the intervention started (pre-treatment), and at 6 and 12 months follow-up measurements. The project and the data collection took place from April 2010 to January 2012.

### Demographic Data

Information on the participants’ employment status, time on ongoing sick leave and level of sick leave was received through registry data from the SSIA. A physician made a classification of the participants’ main problem, based on the stated diagnoses in the sick leave certificates. Data about the participant’s age, educational level and country of birth were obtained through a self-report questionnaire.

### The General Self-Efficacy Scale (GSE)

The GSE assesses individuals’ beliefs in their ability to handle various difficult demands in life [25] and consists of 10 statements rated on a four-point Likert scale from 1 = “Not at all true” to 4 = “Completely true”. Means were calculated as the sum of all answers divided by the number of statements when no more than three statements were missing [26]. In the present study, self-efficacy was normally distributed. The GSE has been validated and translated into Swedish [27].

### The Hospital Anxiety and Depression Scale (HADS)

The HADS consists of two subscales, one for anxiety and one for depression, with seven items each [28]. HADS is responded to on a four-point Likert scale from 0 to 3, and each subscale is summed with a score range from 0 (no distress) to 21 (maximum distress). A score of 0–7 indicated a “non-case”, 8–10 a “possible case” and 11–21 a “probable case” of anxiety or depression. Missing values were handled by replacement of the individual’s mean scores as long as at least four questions for each subscale were answered. HADS has been validated and translated into Swedish [29].

## Statistical Analysis

Descriptive statistics were used for demographics: one-way ANOVAs to determine differences in group means and *χ*^2^ tests for differences in group proportions and for ordinal data.

Linear regression was used to assess associations between independent variables (see below) and the dependent variable self-efficacy at 12 months. Models of adjusted associations between independent variables and self-efficacy at 12 months were performed with multivariate linear regression analyses. Three models were tested: model 1 = group allocation, model 2 = model 1 + age, employment status and time on sick leave, model 3 = model 1 + model 2 and self-efficacy (pre-treatment) and HADS, anxiety and depression (pre-treatment). Dummy variables were created for the two intervention groups: TEAM (TEAM coded as 1 and ACT + control coded as 0) and ACT (ACT coded as 1 and TEAM + control coded as 0). Missing data were handled with multiple imputation (carried out 100 times) for self-efficacy to deal with loss to follow-up, at least one of the three self-efficacy measurement points was required for imputation. This meant that 52 participants were excluded from the analyses based on imputed data. Tests of collinearity indicated no multicollinearity.

Linear mixed models were used for longitudinal analysis of the repeated measurements of self-efficacy, pre-treatment, 6 and 12 months. The purpose of the linear mixed model was to evaluate whether the interventions lead to increased self-efficacy at the 12-month follow-up measurement, taking missing data and all of the three measurement points of self-efficacy into account. The advantage of linear mixed models is that they handle missing data that often arise in longitudinal data and provide the ability to take into account other covariates’ influence on the dependent variable over time [30]. Participants’ ID number was specified as random effect with an unstructured covariance. The analyses were performed adjusting for age, employment status, time on sick leave and HADS, anxiety and depression (pre-treatment). The analyses were based on the intention to treat, i.e. available data for each participant were used in the analyses regardless of their degree of participation in different rehabilitation actions.

In both the linear regression and the linear mixed model country of birth, education level and level of sick leave were not included in the analyses due to the extent of missing values. Associations are presented with 95% confidence intervals (CIs) and B-values. A p value ≤ 0.025 was considered statistically significant. Bonferroni correction was used for pairwise comparisons for two tests that reduced the significance level to a p value ≤ 0.025 (0.05/2 = 0.025). All tests were two-sided.

Data were analysed using the Statistical Package for the Social Sciences, SPSS, version 22 (IBM, Corporation, Armonk, NY, USA).

## Results

### Study Sample

No differences were seen in demographics between the study groups, i.e. TEAM, ACT and control, before the intervention (Table 1) or in self-efficacy at pre-treatment (Table 2). There was some differences between responders and non-responders, where non-responders to the self-efficacy statements in the 12-months follow-up questionnaire had lower self-efficacy at pre-treatment (p = 0.003) and reported higher levels of anxiety (p = 0.004) and depression (p = 0.013) compared to responders. Furthermore, there was a higher proportion of participants being born abroad (p = 0.001) and with full-time sick leave (p = 0.002) among non-responders compared to responders. Three groups of diagnoses were classified: pain (38%), psychiatric (31%) and pain and psychiatric combined (31%).

**Table 1:** Demographics of the study population, by group and total

	TEAM *n* = 161	ACT *n* = 102	Control *n* = 138	Total *n* = 401
Demographic				
Age [mean (SD)]	49.8 (8.6)	47.8 (7.8)	48.1(8.5)	48.7 (8.4)
Country of birth [*n* (%)]				
Sweden	118 (81.9)	70 (80.5)	90 (73.8)	278 (78.8)
Abroad	26 (18.1)	17 (19.5)	32 (26.2)	75 (21.2)
Education level [n (%)]				
Elementary school	25 (17.9)	11 (12.8)	23 (18.9)	59 (17.0)
High school	61 (43.6)	33 (38.4)	46 (37.7)	140 (40.2)
University	35 (25.0)	31 (36.0)	36 (29.5)	102 (29.3)
Other	19 (13.6)	11 (12.8)	17 (13.9)	47 (13.5)
Employment status [n (%)]				
Employed	109 (67.7)	59 (57.8)	88 (63.8)	256 (63.8)
Unemployed	52 (32.3)	43 (42.2)	50 (36.2)	145 (36.2)
Time for sick leave (year), [mean (SD)]	8.2 (3.2)	7.6 (3.1)	7.6 (3.3)	7.8 (3.2)
Level of sick leave [*n* (%)]				
Full-time	75 (52.1)	49 (54.4)	55 (48.7)	179 (51.6)
Part-time	69 (47.9)	41 (45.6)	58 (51.3)	168 (48.4)
HADS [mean (SD)]				
Anxiety	11.0 (5.0)	10.1 (4.9)	11.3 (5.2)	10.9 (5.1)
Depression	9.4 (4.8)	8.4 (4.2)	9.3 (5.1)	9.1 (4.8)

One-way ANOVA were used to investigate differences in means. Chi square tests were used for differences in proportions. No differences were seen between the study groups

**Table 2:** Mean value of self-efficacy over time (pre-treatment, 6 and 12 months), in each group, based on original data and imputed data

	Pre-treatment			6 months			12 months		
	TEAM	ACT	Control	TEAM	ACT	Control	TEAM	ACT	Control
*n*	137	79	121	104	62	93	104	65	85
% missing	14.9	22.5	12.3	35.4	39.2	32.6	35.4	36.3	38.4
mean	2.29	2.42	2.24	2.45	2.47	2.15	2.74	2.61	2.51
SD	0.7	0.7	0.7	0.6	0.6	0.6	0.6	0.7	0.8
Imputed mean	2.29	2.42	2.24	2.41	2.44	2.15	2.68	2.63	2.44

One-way ANOVA were used to investigate differences in means at pre-treatment in the original data. No differences were seen between the study groups

At pre-treatment, the mean value of self-efficacy was 2.30 (range 1–4). All groups increased in self-efficacy from pre-treatment to the 12-month follow-up measurement, with the largest increase for the TEAM group (see Table 2).

### Linear Regression Analyses and Linear Mixed Model

In linear regression model 1, analyses were performed to assess the effect of TEAM and ACT interventions on the dependent variable self-efficacy at 12 months. The TEAM intervention group increased in self-efficacy at 12 months (B = 0.23, 95% CI 0.03–0.44), whereas the ACT intervention had no effect on self-efficacy at 12 months (B = 0.11, 95% CI − 0.12 to 0.34). These results were also found in Model 2; TEAM intervention (B = 0.23, 95% CI 0.03–0.43) and ACT intervention (B = 0.10, 95% CI − 0.12 to 0.33). Similar results were also found in Model 3; TEAM intervention (B = 0.25, 95% CI 0.10–0.41) and ACT intervention (B = 0.02, 95% CI − 0.16 to 0.19). The results in Model 3 also showed that a higher level of self-efficacy at pre-treatment was associated with higher self-efficacy at 12 months (B = 0.68, 95% CI 0.54–0.81).

In the imputed data, TEAM intervention showed increased self-efficacy at 12 months (B = 0.23, 95% CI 0.07–0.38), but ACT intervention showed no effect (B = 0.08, 95% CI − 0.10 to 0.26) in the adjusted analyses (Table 3).

**Table 3:** Linear regressions: associations between group, demographics, self-efficacy at pre-treatment, HADS and self-efficacy at 12 months, B-values and 95% confidence interval (CI) based on original data and imputed data

	Original data				Imputed data			
	Crude	Model 1 *n* = 254	Model 2 *n* = 254	Model 3 *n* = 243	Crude	Model 1	Model 2	Model 3
Group								
TEAM^a^		0.23 (0.03, 0.44)*	0.23 (0.03, 0.43)*	0.25 (0.10, 0.41)*		0.24 (0.05, 0.43)*	0.24 (0.05, 0.43)*	0.23 (0.07, 0.38)*
ACT^b^		0.11 (− 0.12, 0.34)	0.10 (− 0.12, 0.33)	0.02 (− 0.16, 0.19)		0.19 (− 0.02, 0.40)	0.18 (− 0.03, 0.40)	0.08 (− 0.10, 0.26)
Demographics								
Age	0.00 (− 0.01, 0.01)		− 0.01 (− 0.02, 0.00)	0.00 (− 0.01, 0.00)	0.00 (− 0.01, 0.01)		− 0.01, (− 0.02, 0.00)	0.00 (− 0.01, 0.00)
Employment status^c^	0.20 (0.02, 0.38)		0.20 (0.01, 0.39)	0.07 (− 0.07, 0.22)	0.18 (0.01, 0.35)		0.17 (0.00, 0.35)	0.03 (− 0.11, 0.18)
Time on sick leave (y)	− 0.02 (0.04, 0.01)		− 0.01 (− 0.04, 0,01)	− 0.01 (− 0.03, 0.01)	− 0.02 (− 0.04, 0.01)		− 0.01 (− 0.04, 0.01)	− 0.01 (− 0.03, 0.01)
Self-efficacy, pre-treatment	0.71 (0.61, 0.81)*			0.68 (0.54, 0.81)*	0.66 (0.53, 0.78)*			0.59 (0.43, 0.75)*
HADS								
Anxiety	− 0.05 (− 0.07, − 0.04)*			0.01 (− 0.01, 0.03)	− 0.06 (− 0.07, − 0.04)*			0.00 (− 0.02, 0.02)
Depression	− 0.07 (− 0.08, − 0.05)*			− 0.02 (− 0.04, 0.00)	− 0.07 (− 0.08, − 0.05)*			− 0.02 (− 0.04, 0.00)
*Adjusted R* ^2^		0.01	0.03	0.46				

^a^TEAM = 1, ACT + Control = 0

^b^ACT = 1, TEAM + Control = 0

^c^Unemployed (ref.) versus employed

*p ≤ 0.025

The analyses using linear mixed models confirmed the results from the linear regressions. Compared with controls, the TEAM intervention showed significant treatment effects with increases in self-efficacy at the 12-month follow-up (F = 16.43 and p = 0.000). The adjusted analyses also showed that TEAM intervention were associated with higher self-efficacy at 12 months (B = 0.30, 95% CI 0.15–0.44, p = 0.001). No significant effects were found for ACT intervention (B = 0.11, 95% CI − 0.05 to 0.28, p = 0.181) at a 12-month follow-up. The analyses were performed with adjustment for age, employment status, time on sick leave and HADS.

## Discussion

### Main Findings

This study showed that a multidisciplinary team assessment and multimodal intervention increased levels of self-efficacy, over time, compared to the control group. The ACT intervention showed no significant effect on self-efficacy at 12 months compared to the controls. A higher level of self-efficacy at pre-treatment was associated with a higher level of self-efficacy at 12 months.

### Comparisons with Other Studies

In previous analysis using partly the same data set [20], we concluded that participants had lower self-efficacy (mean 2.3) compared to the general population (mean 2.9) [26, 27]. During the intervention period, the women in the TEAM group showed an increased self-efficacy from 2.29 to 2.74, a level that is not far from observed mean values in the general population.

An earlier study by Lagerveld et al. [31] on women on long-term sick leave showed that a higher self-efficacy at baseline was associated with a shorter duration to RTW, and that an increase in self-efficacy during an occupational rehabilitation intervention was a predictor for a shorter time to RTW [31]. In that study, the participants were given CBT to strengthen self-efficacy to support RTW. Beurden et al. [32] found that participants on sick leave that received an intervention based on cognitive behavioural- and problem solving therapy showed increased self-efficacy at the 3-month follow-up. This suggests that it is possible to strengthen self-efficacy with psychological efforts and that improved self-efficacy may be associated with improved work outcomes.

Lorig and co-workers examined a self-management program based on the sources of information for self-efficacy proposed by Bandura (i.e. verbal persuasion, physiological and affective states, vicarious experiences and enactive mastery) [1] for patients with arthritis and fibromyalgia [33]. This study found an increase in self-efficacy at a 1-year follow-up [33]. We believe that these sources of self-efficacy also was applied in our interventions through the designated person (TEAM member for participants in the TEAM group and psychologist for participants in the ACT group), who supported the participants to reach their goals, by making them aware of their own strengths and abilities. This support could be related to verbal persuasion. Furthermore, the participant’s previous work experiences, opportunities for job adaptation (e.g., assisted by the occupational therapist within the TEAM group) and job training would also have been a possible source for strengthening self-efficacy, related to enactive mastery. Finally, an improvement in the participants’ mental and/or physical condition could conceivably lead to an increase in self-efficacy within both intervention groups, and could thus be connected to psychological and affective states as a source for the judgement of self-efficacy [1].

Self-efficacy has been found to be negatively associated with depressive symptoms and anxiety [34], which was also shown in our study. Thus, it is possible that the participants who received psychological treatment with CBT (e.g., ACT) had a reduction of anxiety and depressive symptoms with positive effects on self-efficacy as a consequence. The regulation changes in the social insurance system posed a risk of being outside the compensation system that may have caused stress about the women’s economic situation. This stress may have influenced their self-efficacy negatively. Bandura describes how there is a correlation between the experience of self-efficacy and health, since stress that arises from exposure to events that an individual experiences as uncontrollable can produce biological processes in the body. These biological processes can affect the individual’s health negatively if they are too intensive or prolonged [1]. The support obtained from the interventions may have reduced this stress.

The team members were educated in MI, a conversation method aimed at strengthening individuals’ motivation and commitment to change. MI is client-centered and builds on cooperation between the adviser and the client, based on partnership, acceptance, compassion and evocation [23]. One objective of MI is to increase self-efficacy [35], which has been shown in previous studies, although in areas other than vocational rehabilitation [36, 37]. However, as it is uncertain to what extent MI was applied in the TEAM intervention, it is not possible to know whether MI had any influence in the increase of self-efficacy found for the women in the TEAM group.

Similar to our study, other studies have also shown that it is possible to strengthen self-efficacy in individuals on sick leave. Hees et al. [38] showed that weekly clinical management treatment (including psychoeducation, CBT and supportive therapy) solely, and in combination with occupational therapy (OT) (i.e. sessions with an occupational therapist, meeting with the employer, focus on early RTW, improving work-related coping, increase in self-efficacy to improve communication with stakeholders), showed positive effects on self-efficacy in sick-listed with major depression [38]. Varekamp et al. [39] showed that self-efficacy increased in individuals with chronic physical diseases who took part in a vocational rehabilitation intervention with the aim to support work maintenance and avoid eventual sick leave. The intervention consisted of a group-training program for problem solving in the work situation and intended to increase the individual’s self-awareness and communication skills at work as well as to find solutions. In our study, the TEAM intervention also had a problem-solving approach to support the participants in their rehabilitation and to increase their likelihood for RTW.

In comparison to the above presented studies whose participants had been on sick leave for a maximum of 1 year [31, 32, 38, 39], the participants in the present study had been on sick leave for an extremely long time (a mean time of 7.8 years) and may therefore not be fully comparable to other studies. It is reasonable to assume that the participants who had been on sick leave for such a long time needed a more individual orientation of their rehabilitation (including medical, psychological and social aspects) which the TEAM intervention could provide as opposed to the ACT intervention. This difference between the interventions may also explain the lack of improvement in self-efficacy in the ACT group.

To our knowledge, no studies have investigated the possibility to strengthen self-efficacy for individuals on sick leave for such a long time. Furthermore, previous studies have measured self-efficacy using other scales [31, 32, 34, 38, 39] and in other settings [34, 39]. General self-efficacy, i.e. an individual’s basic belief in their competence to handle a broad variation of demands in different contexts, is a universal construct that is stable [25]. This generality makes the strength of general self-efficacy as it can be used in different domains, in comparison to more specific self-efficacy scales [40]. In addition, we did not explore the association between self-efficacy and RTW, which Lagerveld et al. [31] and Beurden et al. [32] did. They used the RTW self-efficacy scale, a specific scale for measuring the change in self-efficacy associated with RTW, which shows stronger associations with RTW compared to the GSE [18]. Our study showed an increase in general self-efficacy for the women in the TEAM group, although we do not know if they increased their self-efficacy specifically to transition to work.

## Strengths and Limitations

A strength of this study is the randomised controlled design, and the use of official register data and validated assessments of the outcome measurement (self-efficacy). The study also uses time series of data, which made it possible to study longitudinal associations. As always in studies using self-reported data, there is some attrition in the outcome questions. We have however, tried to manage this problem using multiple imputations.

A limitation of this study was that self-efficacy (pre-treatment) and HADS was measured after the randomisation, whereby the scores could already have been influenced by the knowledge of intervention group affiliation. The assessment process changed in the TEAM intervention from assessment by an occupational therapist, psychologist, social worker and a physician (in the first phase) to assessment by physiotherapist and physician (in the second phase), which may be seen as a limitation. However, all the team members were still involved in the team meetings and could contribute to the development of the individual rehabilitation plan.

Finally, there were difficulties determining which of the components in the TEAM intervention that contributed to the increase in self-efficacy over time.

## Conclusion and Implications

The women who participated in this study had been on sick leave for an extremely long time (a mean of 7.8 years) and had a low mean level of self-efficacy prior to inclusion. Despite these circumstances, our study found that a multidisciplinary team assessment and multimodal intervention based on an individual’s needs and taking into account medical, social and psychological aspects, had positive effects on self-efficacy that almost reached the level of the general population. These results imply that self-efficacy needs to be addressed in vocational rehabilitation interventions, at least for women on long-term sick leave because of mental illness and/or pain. Further research should involve a similar intervention, including both men and women, to study which of the components in the intervention that mediate the effects on self-efficacy. In a subsequent step, it would also be valuable to study the association between self-efficacy and RTW.
